# Supplementary material for: The exocyst complex and intracellular vesicles mediate soluble protein trafficking to the primary cilium
Source: Commun Biol. 2024 Feb 21;7:213. doi: 10.1038/s42003-024-05817-2 (PMC10879184; doi:10.1038/s42003-024-05817-2)
Supplement: Supplementary file 2 — Supplementary Figures S1-S6, Supplementary Tables S1-S3 [file 42003_2024_5817_MOESM2_ESM.pdf]

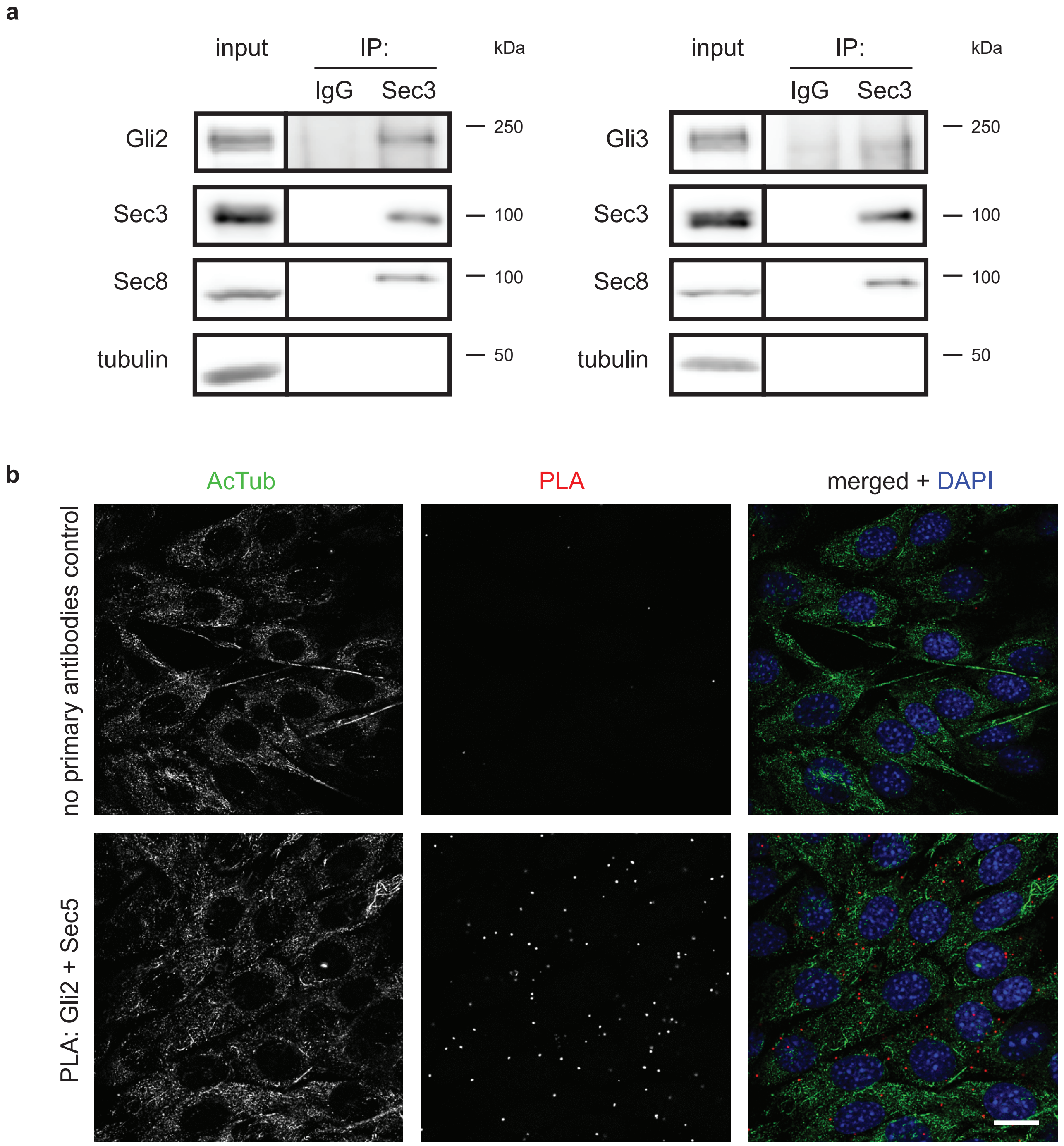

Supplementary Figure S1. **a**, Endogenous Sec3 interacts with Gli2 and Gli3. Cells expressing HA-Gli2(P1-6A) or HA-Gli3 at near-endogenous levels were subjected to co-IP with either anti-Sec3 or species-matched IgG. Eluates were blotted with anti-Gli2 or anti-Gli3, as indicated. Sec8 was used as positive control Sec3 interactor, and tubulin as a negative control. **b**, Proximity Ligation Assay with anti-Gli2 and anti-Sec5 antibodies in NIH/3T3 mouse fibroblasts. Sites of interaction are marked in red. Cilia were stained with anti-acetylated tubulin (green), and nuclei with DAPI (blue). Larger areas with multiple cells are shown compared to Fig. 2B. Scale bar 10μm.

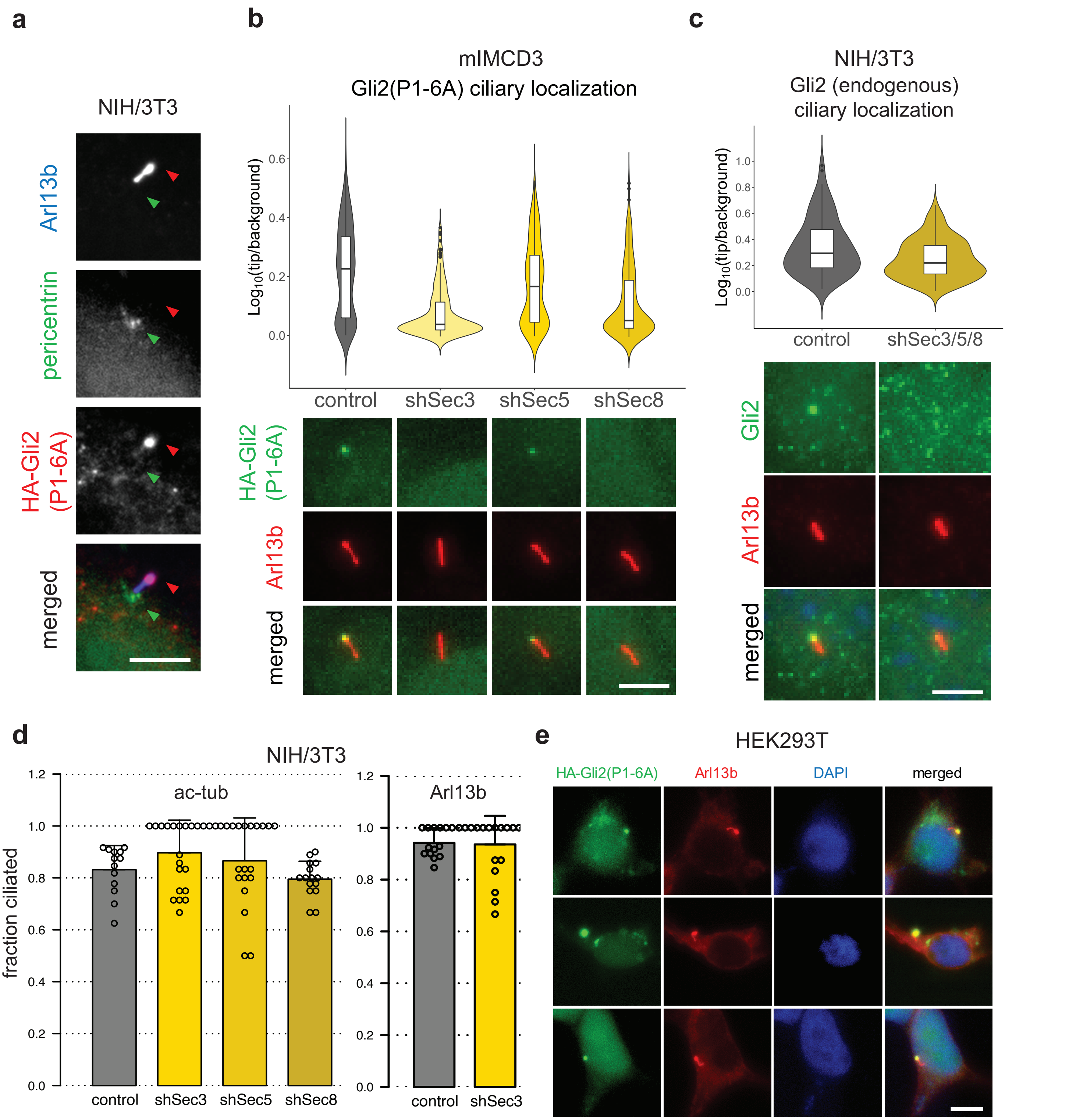

Supplementary Figure S2. **a**, Gli2(P1-6A) accumulates in tips, but not bases, of primary cilia. NIH/3T3 cells stably expressing HA-Gli2(P1-6A) were stained with anti-HA, anti-Arl13b (cilia marker), and anti-pericentrin (basal body/cilia base marker). Gli2 (red arrowhead) strongly accumulates on the opposite side of cilia than pericentrin (green arrowhead). **b**, Effect of Sec3/5/8 shRNA knockdown on relative Gli2(P1-6) ciliary localization in mIMCD3 cells with stable expression of Gli2(P1-6A). Cells were transduced as in Fig. 3a. Control cells were transduced with shRNA against luciferase. Relative localization of Gli2(P1-6A) at the cilium tip was measured as in Fig. 3c for n > 240 cilia per group. Student's t-test control vs shSec3 p-value < 2.2e-16; control vs shSec5 p-value = 0.001585; control vs shSec8 p-value < 2.2e-16. **c**, Relative localization at the cilium tip of endogenous Gli2 in NIH/3T3 cells with shRNA knockdown of Sec3, Sec5, and Sec8 and treated for 24h with SAG agonist. Results are presented as violin plots of log10-transformed ratios of fluorescence intensity of anti-HA staining at cilia tips to the intensity in the surrounding background. Cilia per variant n > 90. Student's t-test analysis control-shSec3/5/8 p-value = 0.0026. Representative images of Gli2 ciliary localization for each condition are presented below. Arl13b was used as a ciliary marker. **d**, Exocyst subunit knockdown does not result in reduced ciliation. Fraction of ciliated cells +/- SD is shown from n>10 images (>100 cells counted per group). Acetylated tubulin (ac-tub; left panel) or Arl13b (right panel) was used as a cilia marker. **e**, HEK293T cells extend primary cilia and accumulate Gli2 at cilia tips. HEK293T cells were transfected with HA-Gli2(P1-6A), starved for 48h in media without serum, and stained for immunofluorescence imaging. Scale bars 5μm.

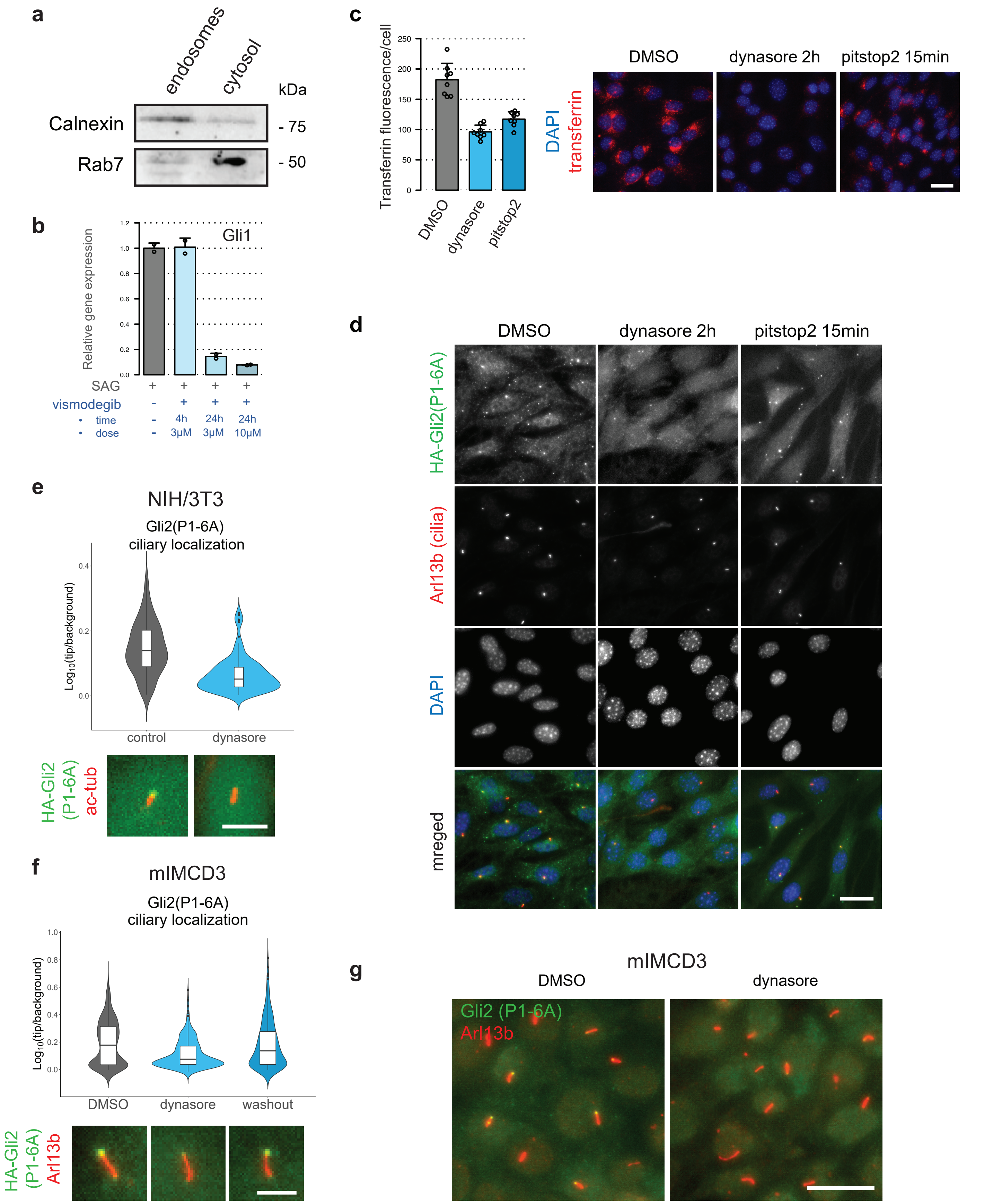

Supplementary Figure S3. **a**, Distribution of ER and late endosome marker between fractions in endosome isolation procedure. Experiment was performed as in Fig. 5C. **b**, The relative mRNA expression level of Gli1 (Hh pathway activity marker) after indicated dose and time of vismogedib treatment. **c**, Dynasore and pitstop2 effectively reduce endocytosis at doses and time points used for cilium Gli2 imaging. Cells were treated as described, followed by incubation with AlexaFluor 647-conjugated transferrin for 20min, washed, fixed, DAPI-stained, and imaged. Average fluorescence signal intensity per cell is shown, with representative images on the right. **d**, Low magnification images of NIH/3T3 cells treated with dynasore or pitstop2 (see Fig. 5d and Fig. 5f). Cell morphology appears normal with no apparent toxicity (cell rounding or detaching, chromatin condensing or fragmenting). **e**, NIH/3T3 Flp-in cells with stable expression of HA-Gli2(P1-6A) were treated with dynasore (24h; 40μM). Relative localization of Gli2(P1-6A) at the cilium tip was measured as in Fig. 3c, except the cilium marker was acetylated tubulin (ac-tub) for n > 100 cilia per group. Student's t-test DMSO vs dynasore p-value = 0. **f**, mIMCD3 Flp-in cells with stable expression of HA-Gli2(P1-6A) were treated with dynasore (2h; 40μM) and then the drug was washed out for 2h. Relative localization of Gli2(P1-6A) at the cilium tip was measured as in Fig. 3c for n > 300 cilia per group. Student's t-test DMSO vs dynasore p-value = 2.433e-13; dynasore vs washout p-value = 2.698e-13; DMSO vs washout p-value = 0.6375. **g**, Dynasore did not disrupt mIMCD3 cells after 2h treatment. Cell morphology and cilia morphology remain unchanged. Scale bars 5μm for **e** and **f**, 20μm for **c**, **d**, and **g**.

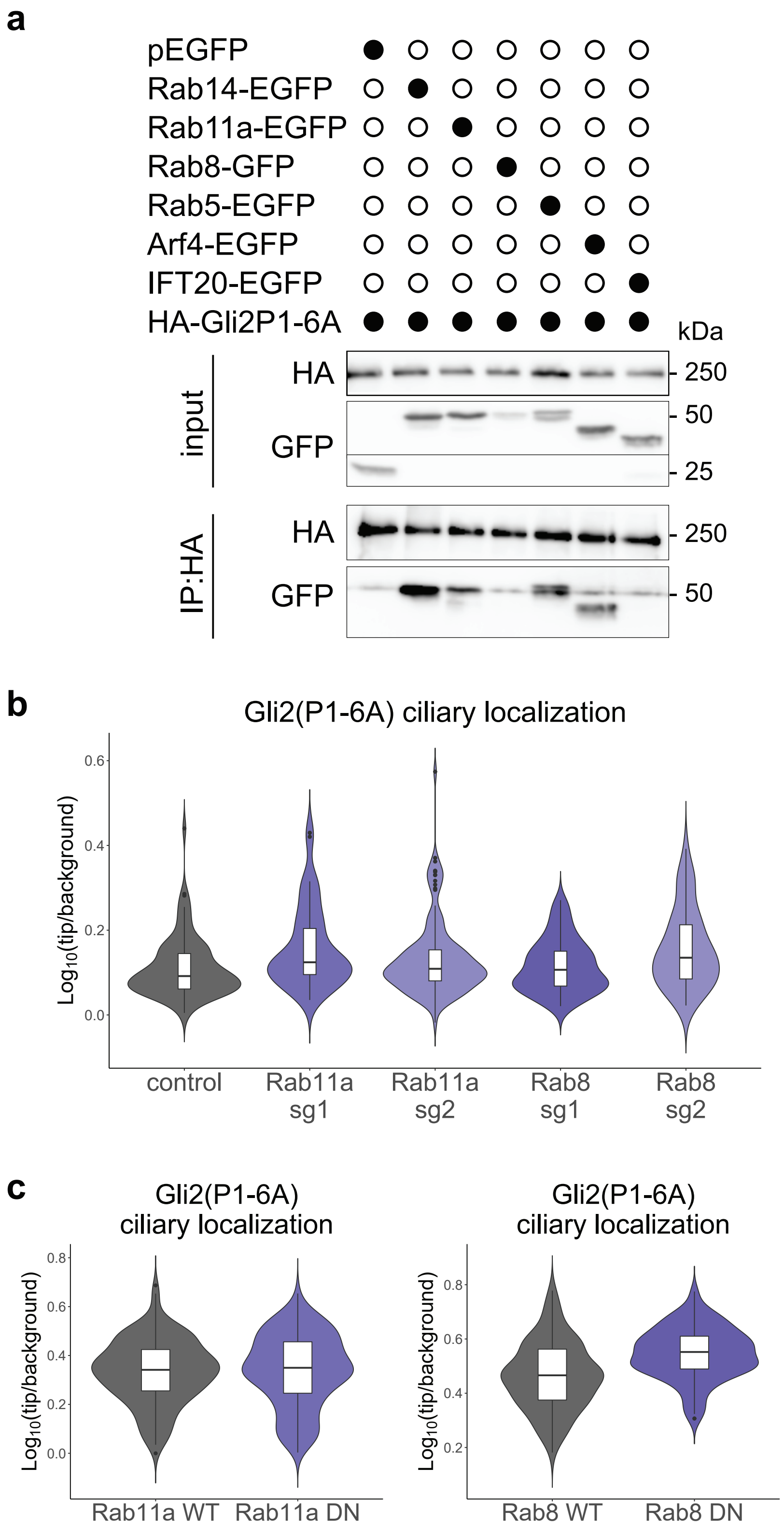

Supplementary Figure S4. **a**, Co-immunoprecipitation of EGFP tagged Rab, Arf, and IFT proteins with HA-Gli2(P1-6A). HEK293T cells were co-transfected with the indicated constructs and co-IP was performed using the HA beads. **b**, Effect of knockout of Rab8 and Rab11a (two sgRNAs each) on Gli2(P1-6A) ciliary localization. Relative localization of Gli2(P1-6A) at the cilium tip was measured as in Fig. 3c for n > 40 cilia per group. Tukey's post-hoc test Rab11a sg1 vs control p-value = 0.01; Rab11a sg1 vs control p-value = 0.01; Rab8 sg1 vs control p-value = 0.99; Rab8 sg2 vs control p-value = 0.01. **c**, Effect of inducible expression of dominant-negative (DN) forms of Rab8 and Rab11a on Gli2(P1-6A) ciliary localization. Relative localization of Gli2(P1-6A) at the cilium tip was measured as in Fig. 3c for n > 120 cilia per group. Student's t-test Rab11a WT vs DN p-value = 0.70; Rab8 WT vs DN p-value = 2.5e-08.

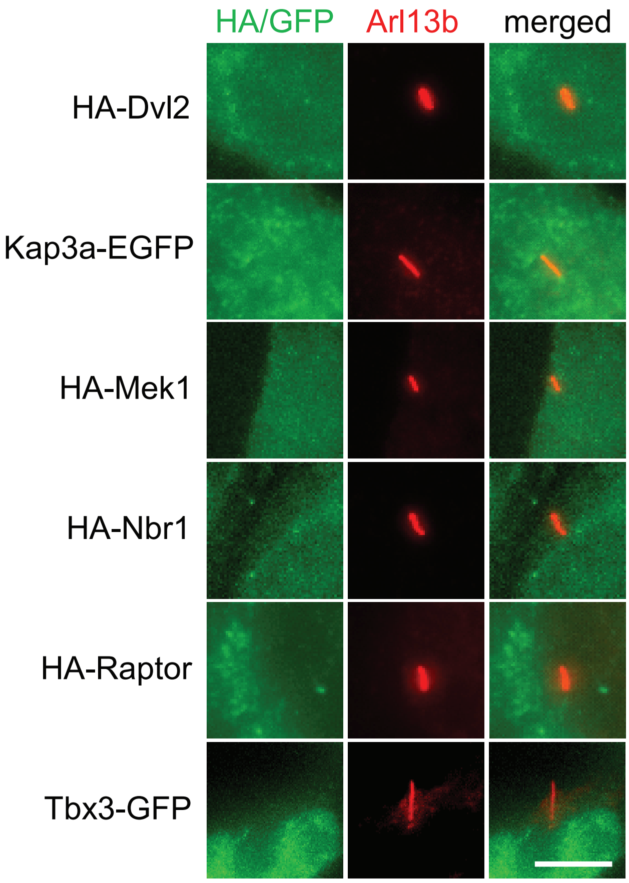

Supplementary Figure S5. Ciliary localization of different putative ciliary proteins we tested in NIH/3T3 cells. Cells were transfected with indicated proteins tagged with HA or GFP and then we observed their ciliary localization. Arl13b was used as a ciliary marker. Scale bar 5μm.

Supplementary Figure S6

Unprocessed blots for Fig. 2a

Gli2

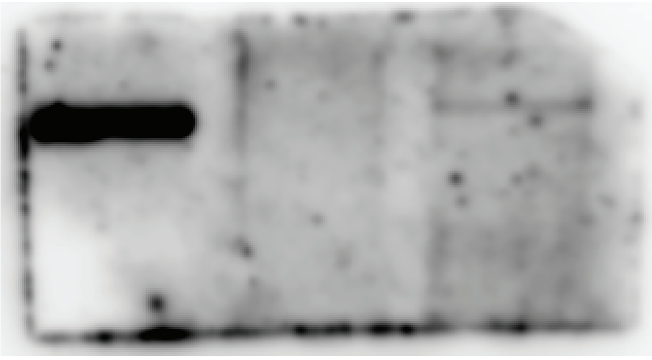

Gli3

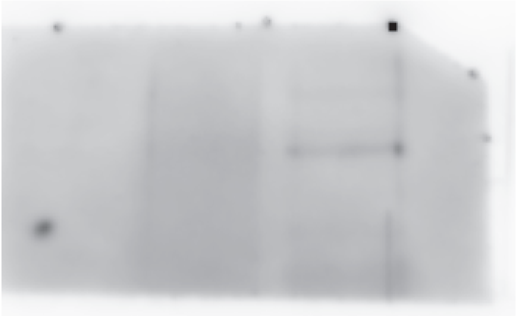

Sec5

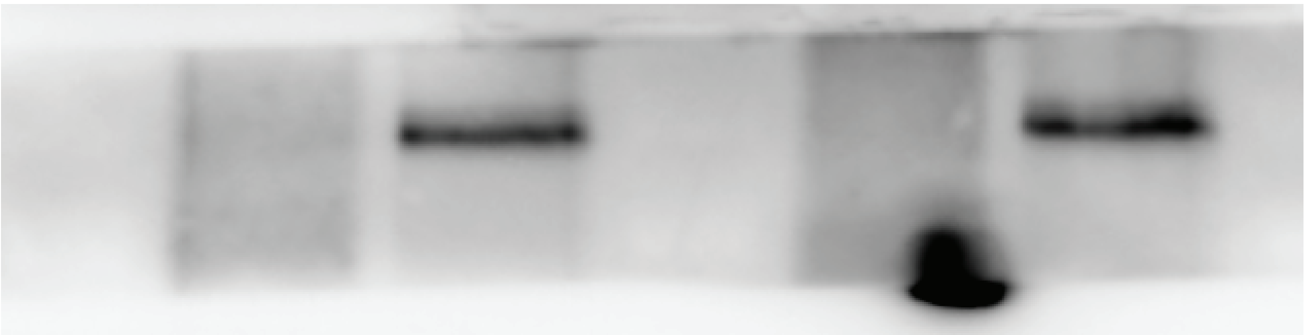

Supplementary Figure S6

Unprocessed blots for Fig. 2c, d, f

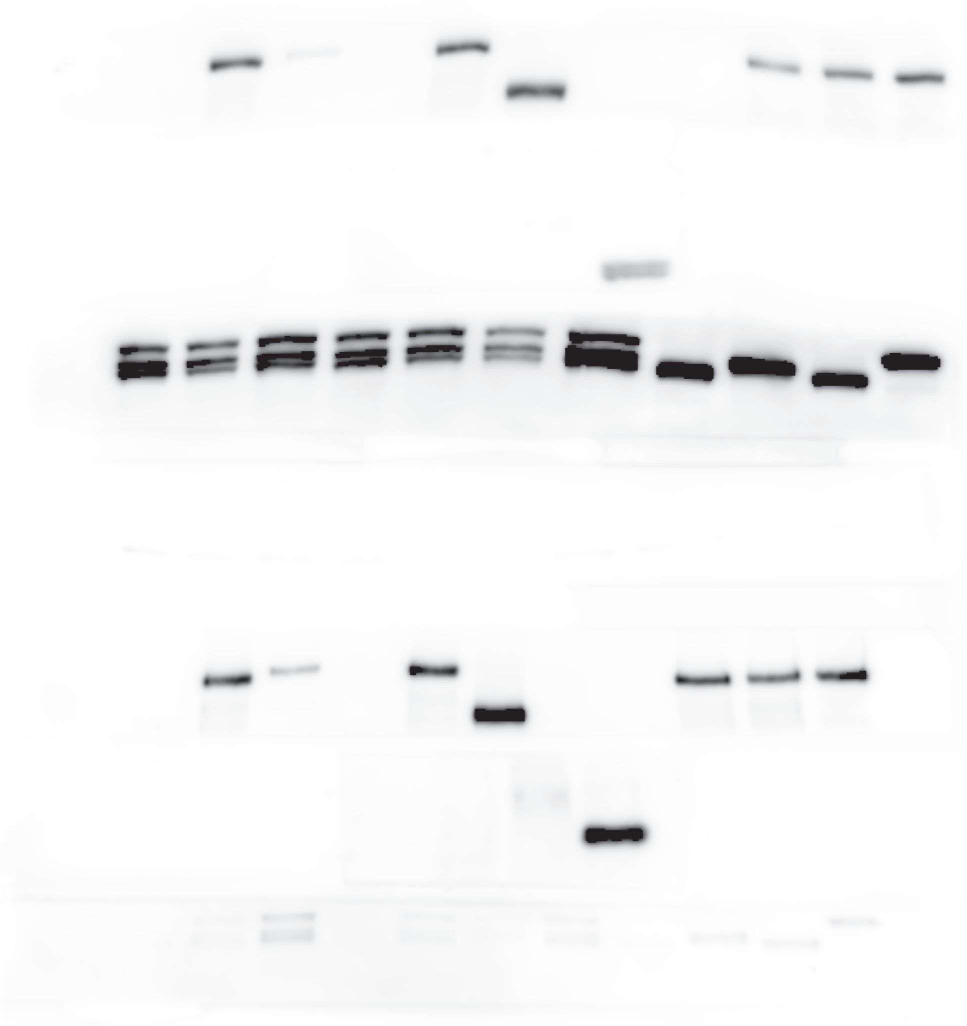

Supplementary Figure S6

Unprocessed blots for Fig. 2e

input HA

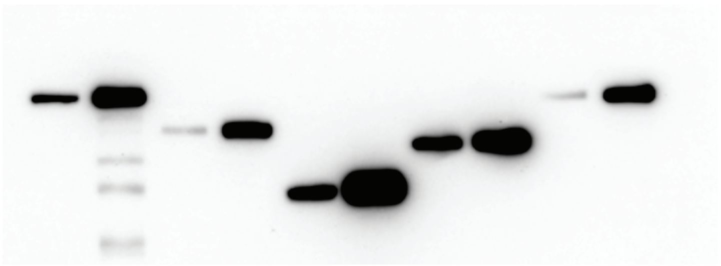

input GFP, actin

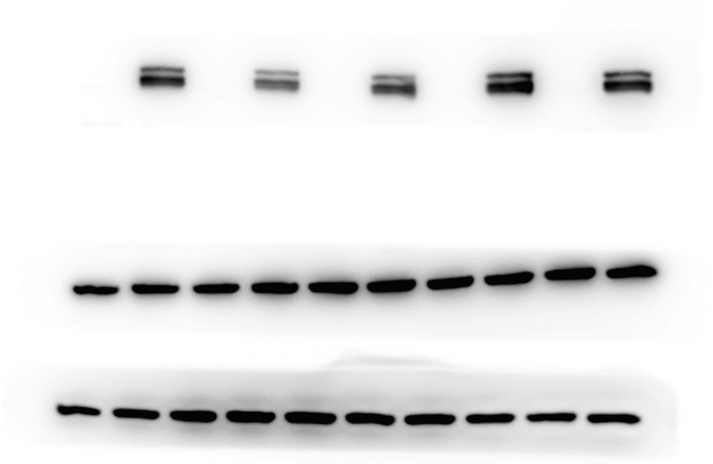

IP HA

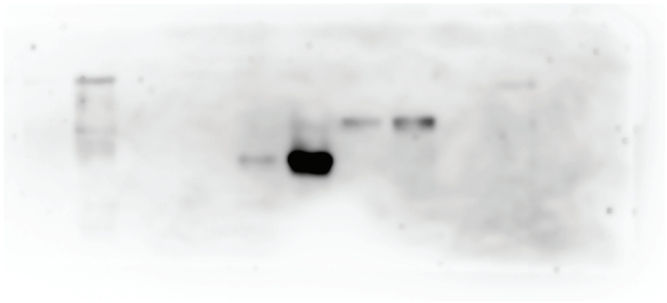

IP GFP

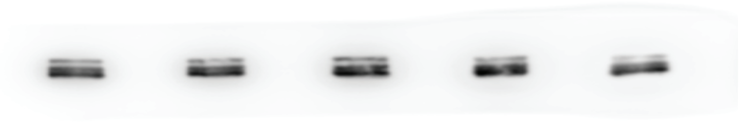

Supplementary Figure S6

Unprocessed blots for Fig. 3a

Sec3

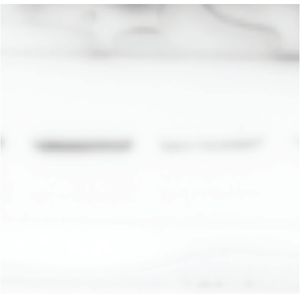

Sec5

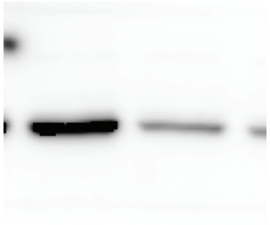

Sec8

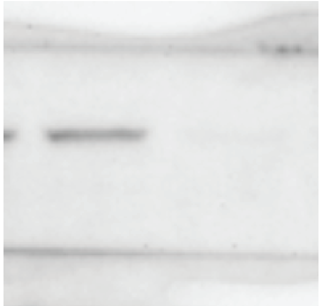

tubulin

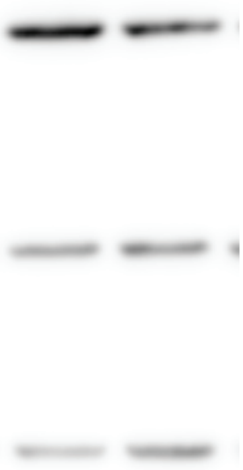

Supplementary Figure S6

Unprocessed blots for Fig. 3b

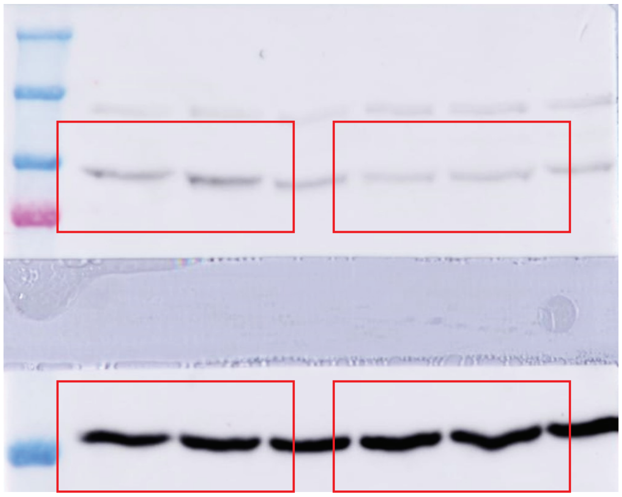

Supplementary Figure S6

Unprocessed blots for Fig. 5c

EEA1, Sec5, ERK1

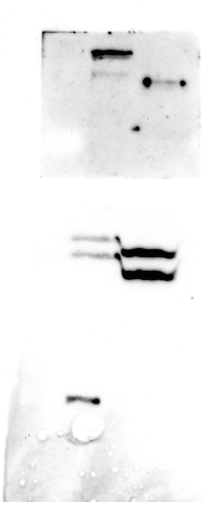

Silver

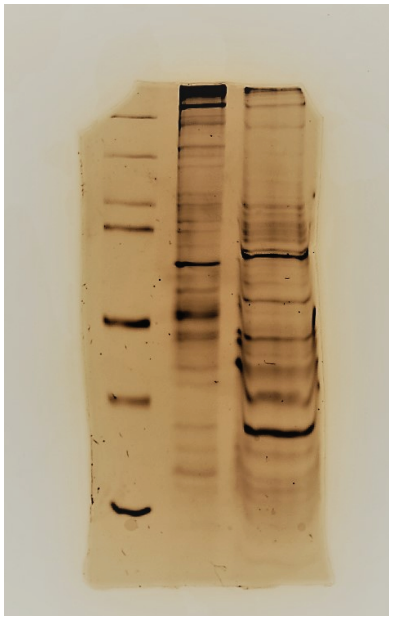

Gli3

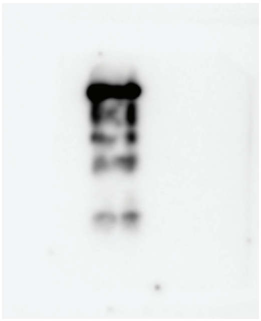

Gli2

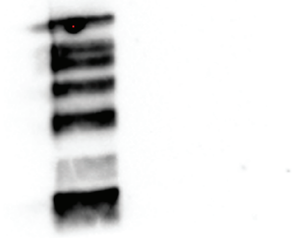

Supplementary Figure S6

Unprocessed blots for Fig. 7a

HA input

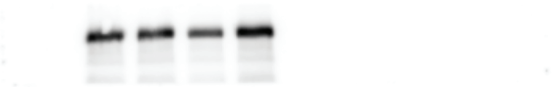

HA iP

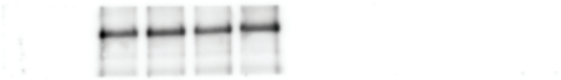

GFP input

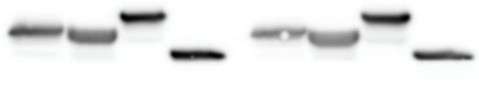

GFP iP

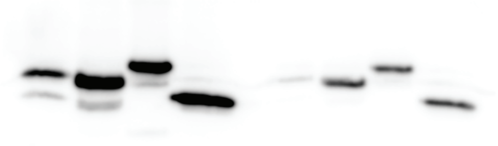

actin

Supplementary Figure S6
Unprocessed blots for Fig. 8d

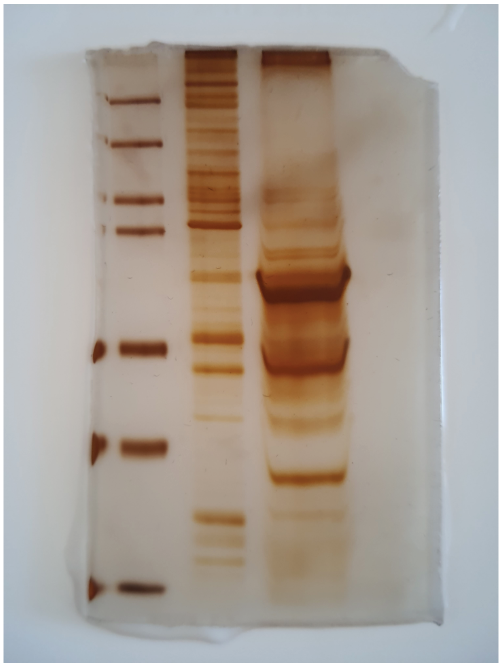

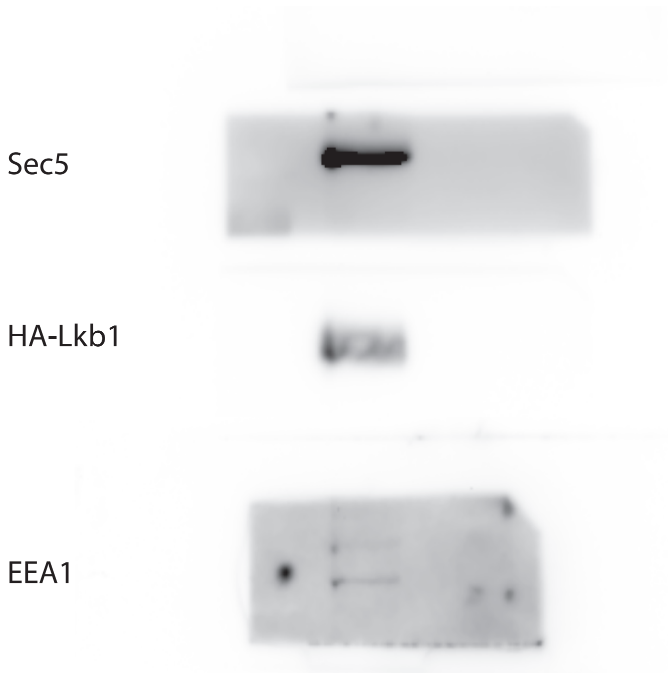

Supplementary Figure S6
Unprocessed blots for Fig. 8e

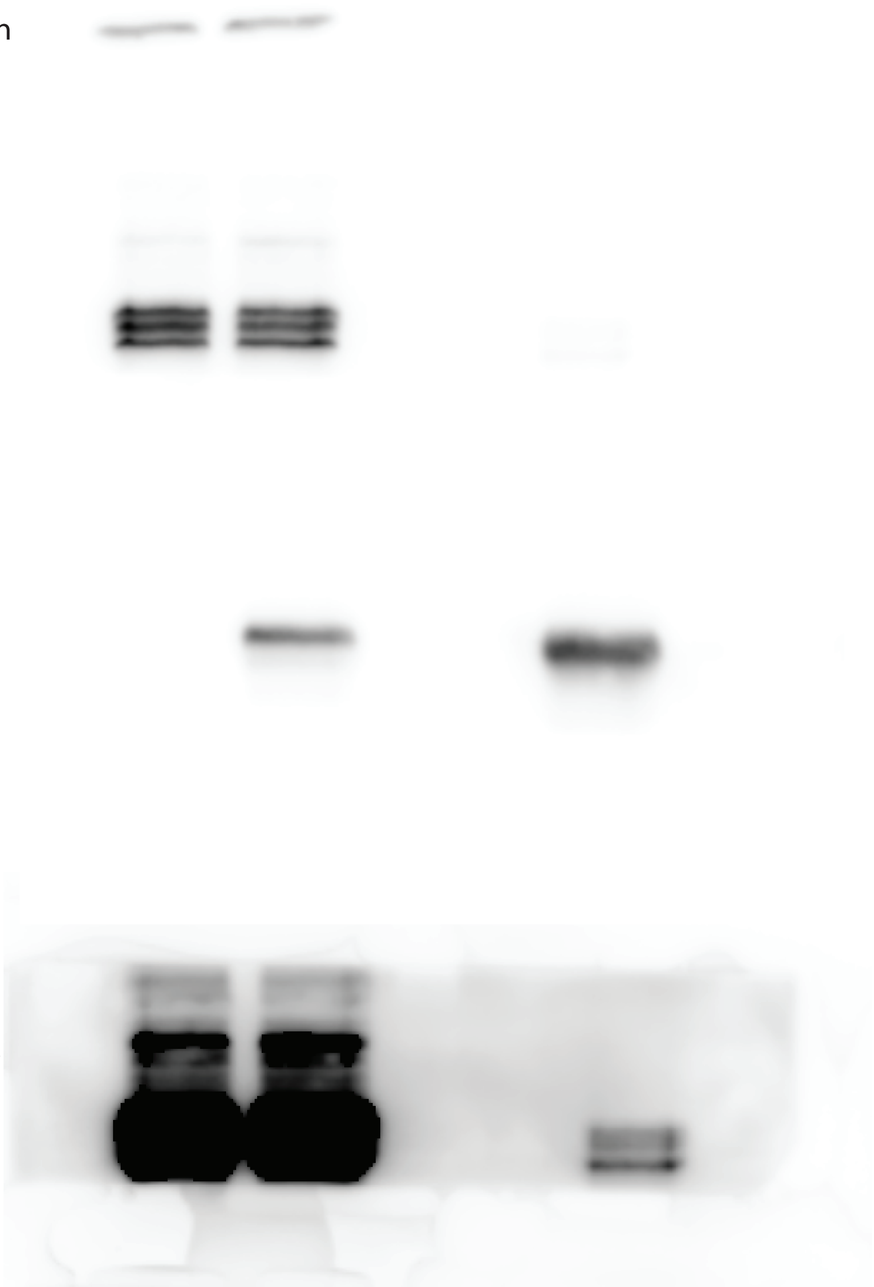

input: actin

input: GFP

input: HA,
IP: HA

input: GFP,
IP: GFP
high
exposure

Supplementary Figure S6

Unprocessed blots for Fig. 8h

GFP-Ubxn10

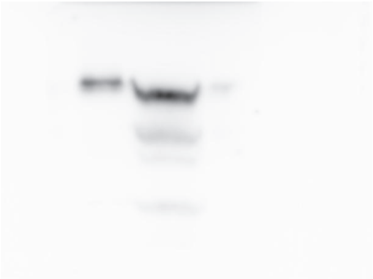

EEA1

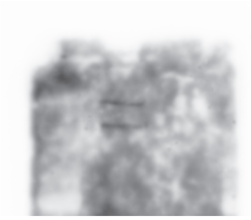

Sec5

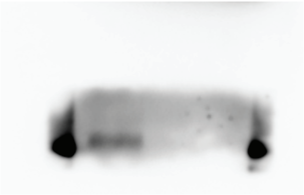

ERK

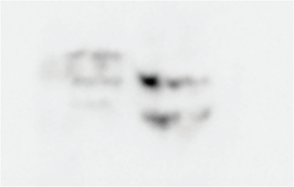

Supplementary Figure S6

Unprocessed blots for Fig. S1a

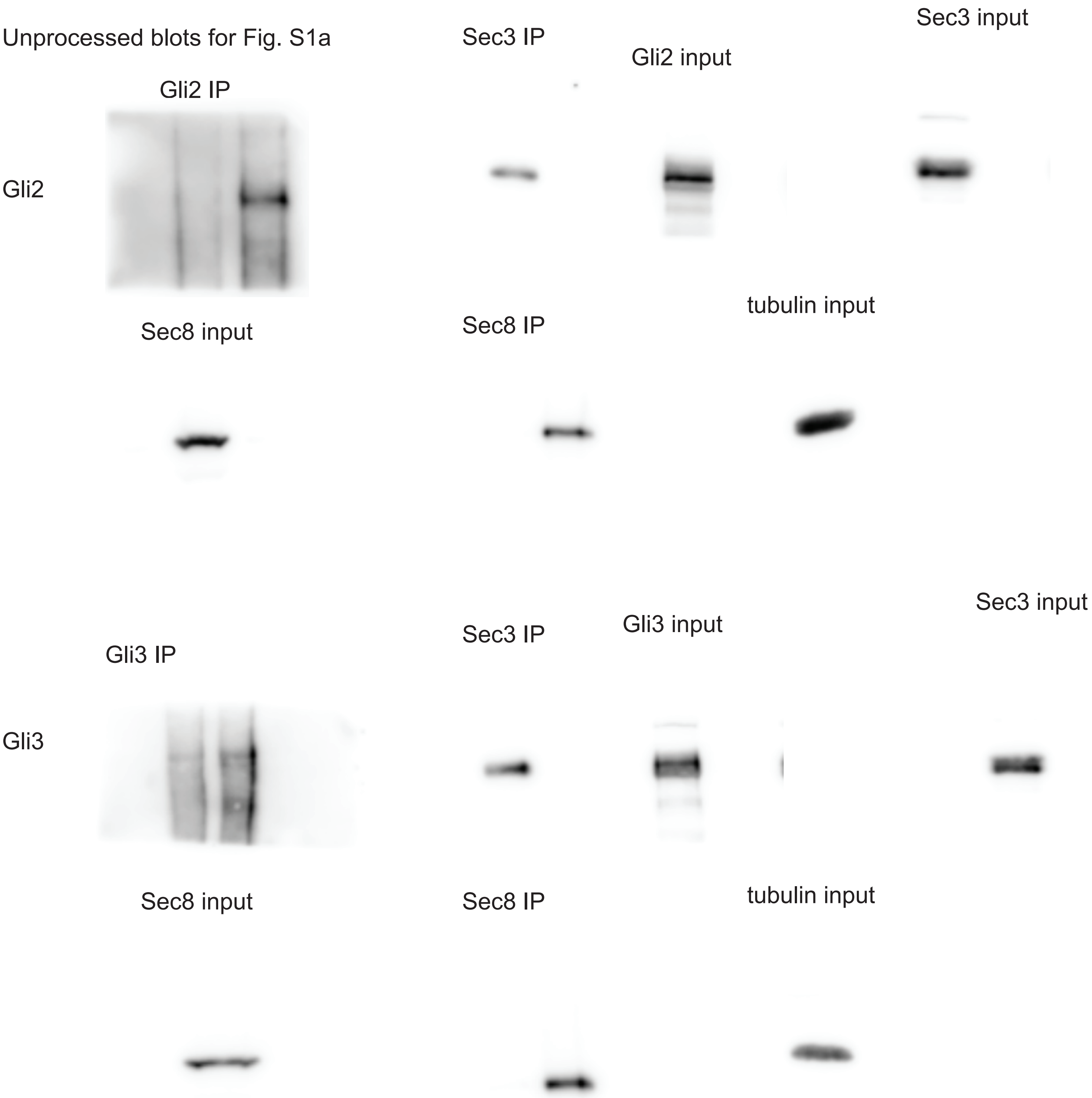

Supplementary Figure S6
Unprocessed blots for Fig. S4a

HA

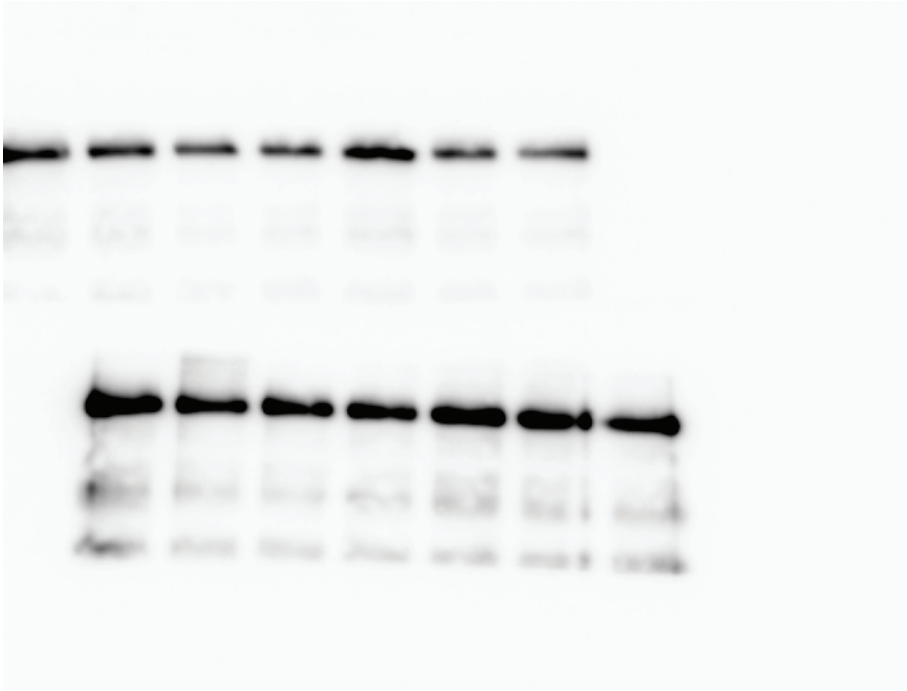

GFP input

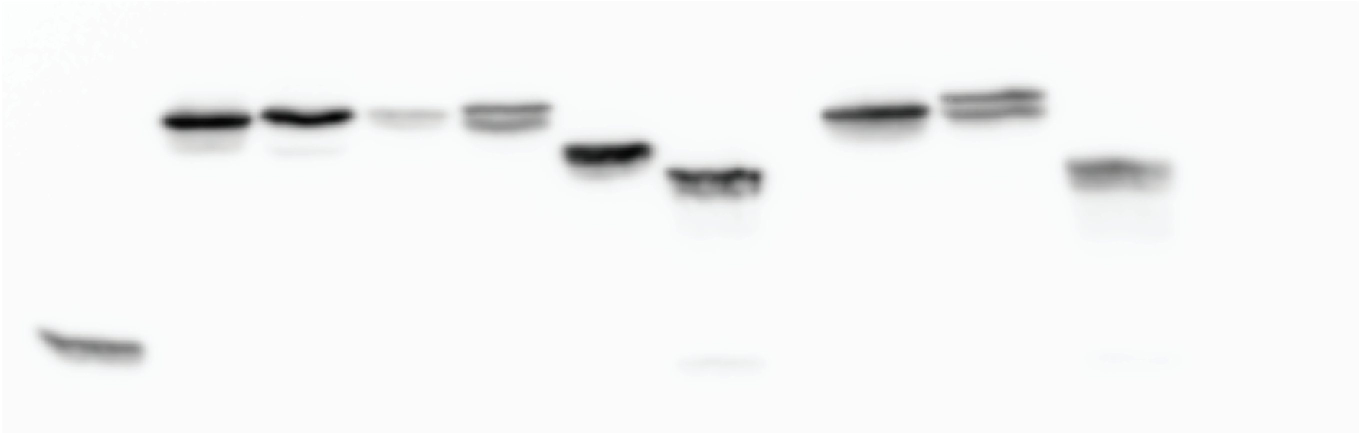

GFP IP

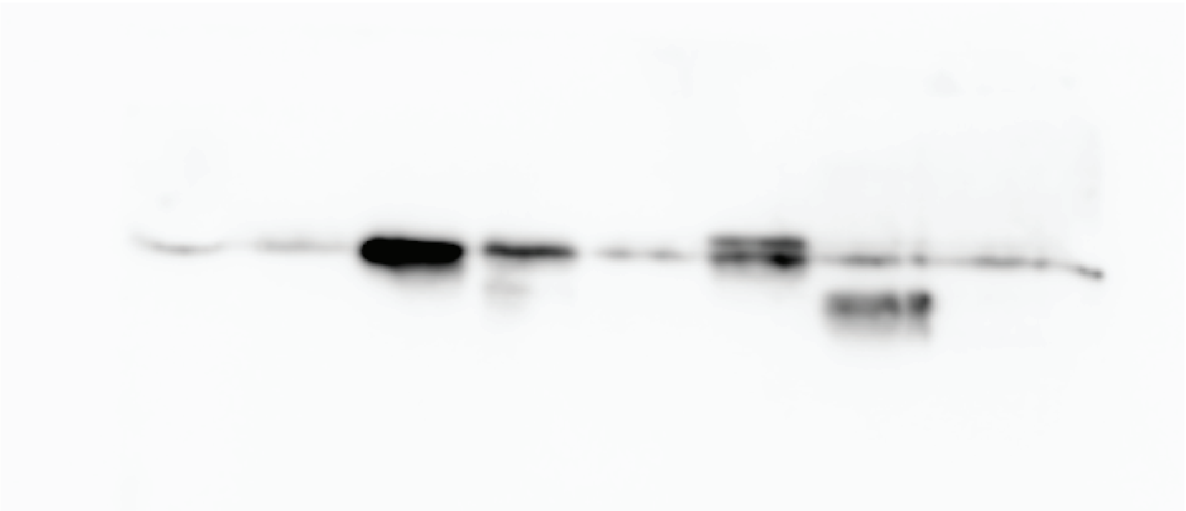

Supplementary Tables S1-S3

Supplementary Table S1. Plasmids used in our experiments ordered from the addgene site

	Plasmid name	Addgene No.	Gift from	References
**1**	pEGFP-C3-Sec3	#53755	Channing Der	[120]
**2**	pEGFP-C3-Sec5	#53756	Channing Der	[120]
**3**	pEGFP-C3-Sec8	#53758	Channing Der	[120]
**4**	pmScarlet_C1	#85042	Dorus Gadella	[121]
**5**	pLKO.1 - TRC cloning vector	#10878	David Root	[122]
**6**	pLKO.1 - blast	#25566	Keith Mostov	[123]
**7**	lentiCas9-Blast	#52962	Feng Zhang	[124]
**8**	LT3GEPIR	#111177	Johannes Zuber	[125]
**9**	lentiGuide-Puro	#52963	Feng Zhang	[124]
**10**	pRSV-Rev	#12253	Didier Trono	[126]
**11**	pMDLg/pRRE	#12251	Didier Trono	[126]
**12**	pMD2.G	#12259	Didier Trono	unpublished
**13**	EGFP-Rab14	#49549	Marci Scidmore	[127]
**14**	EGFP-Rab18	#49550	Marci Scidmore	[127]
**15**	Arf4-GFP	#39556	Paul Melancon	[128]
**16**	pCAG/hArf4(T31N)-HA	#79405	Kazuhisa Nakayama	[129]
**17**	GFP-rab11 DN	#12678	Richard Pagano	[130]
**18**	pGFP-Rab8A	#24898	Maxence Nachury	[131]
**19**	pGFP-Rab8A[T22N]	#24899	Maxence Nachury	[131]
**20**	pLenti-IFT20-EGFP	#118032	Ken Ichi Takemaru	unpublished

Supplementary Table S2. Sequences used for shRNA knockdown, CRISPR edition, and qPCR primers.

name	sequence
shRNA-Sec3	GGAGGTGGACCAGATTGAACT
shRNA-Sec5	GCATACGGCCGAAGAGATAAA
shRNA-Sec8	GCAGGAGCTAAAGCAGATTGT
shRNA-Rab14	CGGTTACACGGAGCTACTATA
shRNA-Rab18	TATCATGGCAGTGAGTATTTG
shRNA-Arf4	CGGTTACACGGAGCTACTATA
shRNA-Luciferase	GCTGGAGAGCAACTGCATAAG
sgRNA Rab23	AAAGACTACAAGAAAACCAT
sgRNA Rab14	CATATAACCACTTAAGCAGC
sgRNA Rab18	ATACTCATCATCGGCGAGAG
sgRNA Arf4	GATCGTGAAAGAATCCAGGA
qPCR Rab14 F	GGTTCAGAGCGGTTACACG
qPCR Rab14 R	TGAGATTCCTTGCGTCTGTC
qPCR Rab18 F	GCACGCAAGCATTCTATGTTG
qPCR Rab18 R	AGCTTGACTCCTTTGTTCTGG
qPCR Arf4 F	AGGATCTGCCAAACGCTATG
qPCR Arf4 R	CCTCATACAGACCAGTTCCTTG
qPCR Sec3 F	TCGCGCTGAGAAAAGATGAC
qPCR Sec3 R	TTCTTGCCAGCTTTGCAGAC
qPCR Sec5 F	CGGAGGTGCAAGTTTTCA AG
qPCR Sec5 R	GCATGGAGGTCGGAAAGA TAC
qPCR Sec8 F	AATTGACCACAGCCATTCGC
qPCR Sec8 R	TCATCCCGTTTGCAATGCAG

Supplementary Table S3. Antibodies used for western blot, immunofluorescence staining, and proximity ligation assay

Antibody	Application	Company	Ref No.
**Primary antibodies**	**Western blot (WB), Immunofluorescence (IF)**		
anti-HA High Affinity	WB (1:1000); IF (1:2000)	Roche	11867423001
anti-HA	WB (1:1000)	BioLegend	901501
anti-GFP	WB (1:1000)	Genetex	GTX113617
anti-Arl13b	IF (1:2000)	Proteintech	17711-1-AP
anti-Sec5	WB (1:500); IF (1:200); PLA	Proteintech	12751-1-AP
anti-Sec5	WB (1:2000)	Proteintech	66011-1-Ig
anti-Sec3	WB (1:1000); IF (1:500)	Proteintech	11690-1-AP
anti-Sec8	WB (1:300)	Proteintech	11913-1-AP
anti-α-tubulin	WB (1:1000)	Sigma	T6199
anti-beta-actin	WB (1:1000)	Sigma	A1978-100UL
anti-Gli2	WB (1:1000)	home-made by Davids biotechnologie	
anti-Gli2	PLA	R&D Systems	AF3635
anti-Gli3	WB (1:1000)	R&D Systems	AF3690
anti-Pericentrin	IF (1:200)	BD Biosciences	611814
anti-EEA1	WB (1:1000)	BD Biosciences	610456
anti-acetylated tubulin	IF (1:1000)	Sigma	T6793
**Secondary antibodies**			
anti-mouse alexa-488	IF (1:1000)	Jackson Immunoresearch	715-545-151
anti-rabbit alexa-488	IF (1:1000)	Jackson Immunoresearch	711-545-152
anti-rat alexa-488	IF (1:1000)	Jackson Immunoresearch	712-545-153
anti-rabbit alexa-Cy3	IF (1:1000)	Jackson Immunoresearch	711-165-152
anti-mouse alexa-594	IF (1:1000)	Jackson Immunoresearch	715-585-151
anti-rabbit alexa-594	IF (1:1000)	Jackson Immunoresearch	711-585-152
anti-rat alexa-647	IF (1:1000)	Jackson Immunoresearch	712-605-153
HRP anti-mouse	WB (1:2500)	BioLegend	405306
HRP anti-rabbit	WB (1:2500)	BioLegend	406401
HRP anti-goat	WB (1:2500)	Sigma	A5420

References

120. Martin, T. D. *et al.* Ral and Rheb GTPase activating proteins integrate mTOR and GTPase signaling in aging, autophagy, and tumor cell invasion. *Mol. Cell* **53**, 209–220 (2014).

121. Bindels, D. S. *et al.* mScarlet: a bright monomeric red fluorescent protein for cellular imaging. *Nat. Methods* **14**, 53–56 (2017).

122. Moffat, J. *et al.* A lentiviral RNAi library for human and mouse genes applied to an arrayed viral high-content screen. *Cell* **124**, 1283–1298 (2006).

123. Bryant, D. M. *et al.* A molecular network for de novo generation of the apical surface and lumen. *Nat. Cell Biol.* **12**, 1035–1045 (2010).

124. Sanjana, N. E., Shalem, O. & Zhang, F. Improved vectors and genome-wide libraries for CRISPR screening. *Nat. Methods* **11**, 783–784 (2014).

125. Fellmann, C. *et al.* An optimized microRNA backbone for effective single-copy RNAi. *Cell Rep.* **5**, 1704–1713 (2013).

126. Dull, T. *et al.* A third-generation lentivirus vector with a conditional packaging system. *J. Virol.* **72**, 8463–8471 (1998).

127. Huang, B. *et al.* The Anaplasma phagocytophilum-occupied vacuole selectively recruits Rab-GTPases that are predominantly associated with recycling endosomes. *Cell. Microbiol.* **12**, 1292–1307 (2010).

128. Chun, J., Shapovalova, Z., Dejgaard, S. Y., Presley, J. F. & Melançon, P. Characterization of class I and II ADP-ribosylation factors (Arfs) in live cells: GDP-bound class II Arfs associate with the ER-Golgi intermediate compartment independently of GBF1. *Mol. Biol. Cell* **19**, 3488–3500 (2008).

129. Nakai, W. *et al.* ARF1 and ARF4 regulate recycling endosomal morphology and retrograde transport from endosomes to the Golgi apparatus. *Mol. Biol. Cell* **24**, 2570–2581 (2013).

130. Choudhury, A. *et al.* Rab proteins mediate Golgi transport of caveola-internalized glycosphingolipids and correct lipid trafficking in Niemann-Pick C cells. *J. Clin. Invest.* **109**, 1541–1550 (2002).

131. Nachury, M. V. *et al.* A core complex of BBS proteins cooperates with the GTPase Rab8 to promote ciliary membrane biogenesis. *Cell* **129**, 1201–1213 (2007).
