# Supplementary material for: The exocyst complex and intracellular vesicles mediate soluble protein trafficking to the primary cilium
Source: Commun Biol. 2024 Feb 21;7:213. doi: 10.1038/s42003-024-05817-2 (PMC10879184; doi:10.1038/s42003-024-05817-2)
Supplement: Supplementary file 3 — Description of Additional Supplementary Files [file 42003_2024_5817_MOESM3_ESM.pdf]

Description of Additional Supplementary Files

File name: Supplementary Data 1
Description: Proteins identified in Gli3 co-IP/MS in cytoplasmic and nuclear fractions of NIH/3T3 cells treated with the Smoothened agonist SAG.

File name: Supplementary Data 2
Description: Proteins identified in HA-Gli2 P1-6A co-IP/MS in control NIH/3T3 Flp-In cells and cells expressing dominant negative Kif3a (dnKif3a).

File name: Supplementary Data 3
Description: Individual data points for bar plots and violin plots.
